# *In Vivo* and *in vitro* antitumor activity of tomatine in hepatocellular carcinoma

**DOI:** 10.3389/fphar.2022.1003264

**Published:** 2022-09-09

**Authors:** Cesar Echeverría, Aldo Martin, Felipe Simon, Cristian O. Salas, Mariajesus Nazal, Diego Varela, Ramón A. Pérez-Castro, Juan F. Santibanez, Ricardo O. Valdés-Valdés, Oscar Forero-Doria, Javier Echeverría

**Affiliations:** ^1^ Facultad de Medicina, Universidad de Atacama, Copiapó, Chile; ^2^ Faculty of Life Science, Universidad Andres Bello, Santiago, Chile; ^3^ Millennium Nucleus of Ion Channel-Associated Diseases (MiNICAD), Santiago, Chile; ^4^ Millennium Institute on Immunology and Immunotherapy, Santiago, Chile; ^5^ Departamento de Química Orgánica, Facultad de Química y de Farmacia, Pontificia Universidad Católica de Chile, Santiago, Chile; ^6^ Programa de Fisiología y Biofísica, Instituto de Ciencias Biomédicas, Facultad de Medicina, Universidad de Chile, Santiago, Chile; ^7^ In vivo Tumor Biology Research Facility, Centro Oncológico, Universidad Católica Del Maule, Talca, Chile; ^8^ Laboratorio de Investigaciones Biomédicas, Facultad de Medicina, Universidad Católica Del Maule, Talca, Chile; ^9^ Group for Molecular Oncology, University of Belgrade, Institute for Medical Research, National Institute of Republic of Serbia, Belgrade, Serbia; ^10^ Centro Integrativo de Biología y Química Aplicada (CIBQA), Universidad Bernardo O’Higgins, Santiago, Chile; ^11^ Departamento de Ciencias Del Ambiente, Facultad de Química y Biología, Universidad de Santiago de Chile, Santiago, Chile

**Keywords:** Solanum glycoalkaloids, tomatine, antitumoral activity, hepatocellular carcinoma, apoptosis, caspase pathways

## Abstract

**Background:** There is abundant ethnopharmacological evidence the uses of regarding *Solanum* species as antitumor and anticancer agents. Glycoalkaloids are among the molecules with antiproliferative activity reported in these species.

**Purpose:** To evaluate the anticancer effect of the *Solanum* glycoalkaloid tomatine in hepatocellular carcinoma (HCC) *in vitro* (HepG2 cells) and *in vivo* models.

**Methods:** The resazurin reduction assay was performed to detect the effect of tomatine on cell viability in human HepG2 cell lines. Programmed cell death was investigated by means of cellular apoptosis assays using Annexin V. The expression of cancer related proteins was detected by Western blotting (WB). Reactive oxygen species (ROS) and calcium were determined by 2,7-dichlorodihydrofluorescein diacetate and Fluo-4, respectively. Intrahepatic HepG2 xenograft mouse model was used to elucidate the effect of tomatine on tumor growth *in vivo*.

**Results and Discussion:** Tomatine reduced HepG2 cell viability and induced the early apoptosis phase of cell death, consistently with caspase-3, -7, Bcl-2 family, and P53 proteins activation. Furthermore, tomatine increased intracellular ROS and cytosolic Ca^+2^ levels. Moreover, the NSG mouse xenograft model showed that treating mice with tomatine inhibited HepG2 tumor growth.

**Conclusion:** Tomatine inhibits *in vitro* and *in vivo* HCC tumorigenesis in part via modulation of p53, Ca^+2^, and ROS signalling. Thus, the results suggest the potential cancer therapeutic use of tomatine in HCC patients.

## Introduction

Wild species of genus *Solanum* (Solanaceae) are characterized by the production of several types of glycoalkaloids (GA), among them tomatine. Tomatine has been detected in leaves and tubers of species of *Solanum* genus, endemic of western South America such as *Solanum acaule* Bitter., *S. curtilobum* Juz. and Bukasov, *S. demissum* Lindl., *S. microdontum* Bitter, *S. polyadenium* Greenm., and *S. tarijense* Hawkes, as well as only in the leaves of *S. chomatophilum* Bitter, *S. raphanifolium* Cárdenas and Hawkes, *S. tuberosum* ssp. *andigena* (Juz. and Bukasov) Hawkes (white), *S. tuberosum* ssp. *andigena* (Juz. and Bukasov) Hawkes (violet), and *S. tubersoum* ssp. *andigena* (Juz. and Bukasov) Hawkes (Distl and Wink, 2009). It also is found in *S. brevidens* L., *S. commersonii* Dun., *S. etuberosum* L., *S. jamesii* L., and *S. pinnatisectum* L. (Osman et al., 1978; Gregory et al., 1981; Van Gelder, 1985). There is abundant ethnopharmacological evidence regarding the uses of these species as antitumor and anticancer agents (Kaunda and Zhang, 2019). Tomatine, one of the glycoalkaloids in *Solanum* species, has beneficial bioactivities, including antibiotic, anti-inflammatory, antioxidant, antimalarial, antifungal, cholesterol-lowering, cardiovascular, and immunological effects (Friedman, 2013).

Recently published evidence suggests that tomatine presents antitumor activity, as demonstrated by several *in vitro* and *in vivo* studies (Faria-Silva et al., 2022). For instance, *in vitro* studies have shown that tomatine is highly effective in inhibiting the growth of human cancer cell lines of the breast (Choi et al., 2012; Shi et al., 2013), colon (Kim et al., 2015; Rudolf and Rudolf, 2016), gastric (Xiuwei et al., 2007; Choi et al., 2012), hepatocellular (Xiuwei et al., 2007; Del Giudice et al., 2015), prostate (Choi et al., 2012; Huang et al., 2015), and ovarian (Wu et al., 2021) among others. At the same time, *in vivo* experiments indicate carcinomas that tomatine showed great antitumor potential in mice mammary adenocarcinoma (Tomsik et al., 2013), and HL60 xenograft tumor (Chao et al., 2012); in rainbow trout stomach tumors (Friedman et al., 2007); and subcutaneous and orthotopic xenograft tumors of human prostate cancer PC-3 cells (Lee et al., 2013a; Lee et al., 2013b, Lee et al., 2022).

The incidence and mortality of hepatocellular carcinoma (HCC) have increased in North America and several European regions and declined in traditionally high-risk regions, including Japan and parts of China (Kulik and El-Serag, 2019). Specifically, the *in vitro* cytotoxic effect of tomatine in HepG2 cells produced an inhibition with an IC_50_ of 43 μg/ml (Friedman, 2013). Correspondingly, tomatine, in a range of 0.1–100 μg/ml, has proven to be a potent dose-dependent inhibitor, reducing the growth of liver cancer HepG2 cell lines from 46.3 to 89.2%. Also, 1 μg/ml of tomatine showed better anticarcinogenic activity against human liver cancer cells than doxorubicin (Lee et al., 2004). Moreover, cytotoxicity analysis of α-tomatine showed that it induced a significant cytotoxic effect on both normal liver cells and liver cancer cells (Del Giudice et al., 2015). In turn, *in vivo* analysis support confirm the health-promoting effects of tomatine in animal models (Friedman, 2013). For instance, long-term experiments in rainbow trout showed that administration of tomatine (2000 ppm) and dibenzo [*a,l*]pyrene (DBP) (224 ppm) reduced incidence of liver tumors the induce by DBP (Friedman et al., 2007).

Although tomatine exerts antitumor functions on hepatic tumor cells, the molecular and biological mechanisms are not well elucidated so far. Also, the tomatine *in vivo* antitumor activity has not yet been addressed in mammalian models. This study aims to 1) determine whether tomatine induces HCC cell death 2) elucidate the involved molecular pathways that may explain the antiproliferative activity and, 3) assess is inhibited whether HCC cell tumor growth in a xenograft animal model.

Using HepG2 cells as the HCC model, the results indicated that tomatine induced inhibition of cell proliferation and promoted apoptosis mainly via regulation of apoptotic mediators and p53 activation. These results were along with the increased intracellular reactive oxygen species (ROS) and cytosolic Ca^+2^. Also, tomatine reduced HepG2 tumor growth in a mice xenograft model, suggesting this agent’s potential use in anticancer therapies.

## Materials and methods

### Chemical and reagents

Tomatine (purity >98%; CAS: 17406-45-0) was purchased from Sigma Chemical Co. (St. Louis, MO, United States). Doxorubicin hydrochloride (CAS: 25316-40-9), ionomycin (CAS: 56092-81-0), resazurin (7-hydroxy-10-oxidophenoxazin-10-ium-3-one; CAS: 550-82-3) and 2,7-dichlorodihydrofluorescein diacetate (DCFH-DA; CAS: 4091-99-0) were purchased from Sigma-Aldrich (Santiago, Chile). The Fluo-3AM (CAS: 121714-22-5) was obtained from Biovision (San Francisco, CA). All other reagents were of analytical grade and were purchased from Sigma (United States) and Merck (Germany).

### Cell culture and treatments

The human hepatocellular carcinoma HepG2 cell line (American Type Culture Collection HB-8065) was cultured with Dulbecco’s Modified Eagle Medium (DMEM) supplemented with 10% fetal bovine serum (FBS) (Gibco, NY, United States) and antibiotic-antimycotic mixture (Gibco, NY, United States) at 37°C in a humidified 5% CO_2_ incubator.

### Cell viability assay

HepG2 viability was determined using the resazurin assay as previously described (Riss et al., 2017). Briefly, exponentially growing cells were seeded in 96 well plates in a density of 5 × 10^3^ cells/well and incubated with 100 ml per well culture media overnight. Then, culture media was replaced with 100 ml fresh media containing tomatine treatments at indicated concentrations. After 24 h of treatment, 20 ml resazurin at a concentration of 0.15 mg/ml in PBS was added to each of the wells and then incubated at 37°C for an additional 4 h. Next, the absorbance signal was quickly measured at 570/600 nm (excitation/emission wavelengths).

### Apoptosis assays

To determine the capacity of tomatine to induce cellular apoptosis, the activity of the key effector caspases 3 and 7 by a Caspase-Glo 3/7 assay (Promega Madison, WI, United States) was determined. Cells were seeded at a density of 2000 cells/well in 96-well white-walled plates. The next day, cells were treated with 5 µM of tomatine. Cleaved caspase 3/7 activity was assessed after 4 and 8 h. Caspase-Glo reagent was added into the wells, carefully mixed, and incubated in the dark for 90 min at room temperature. Absorbance and luminescence signals were measured using a microplate reader (Tecan infinite^®^ M200 Pro, Männedorf, Switzerland). Each treatment was read in at least four replicates.

Furthermore, Apoptosis was evaluated through flow cytometry analysis by detecting extracellular membrane phosphatidylserine expression on early apoptotic cells using “Alexa Fluor 488 Annexin V/Dead Cell Apoptosis” Kit; the protocol was carried out as described by the manufacturer’s instructions (Invitrogen, Carlsbad, CA, United States). Cells were analyzed in a flow cytometer model BD FACS Canto II (BD Biosciences, United States), and 10,000 events were analyzed by sample.

### Western blot

The western blot method used was previously described by our research group (Echeverría et al., 2015). For a detailed list of antibodies used, see Supplementary Table S1.

### Fluorescence microscopy

The immunofluorescence assay was conducted as previously reported by our research group (Echeverría et al., 2015). The antibodies’ specific details are described in Supplementary Table S2. A microscopy EVOS^®^ FLoid^®^ cell (Life Technologies, Carls-bad, CA, United States) was used to record and analyze immune-stained cells.

### Determination of intracellular reactive oxygen species

HepG2 cells with or without treatment were subjected to a DCFH-DA assay to determine the intracellular ROS levels. The generated intracellular DCF fluorescence was quantified using a fluorescence plate reader (Tecan infinite^®^ M200pro). Intracellular ROS were expressed as relative fluorescence units (RFU).

### Intracellular Ca^2+^ assays

Plated HepG2 cells were mounted in a perfusion chamber on the stage of an inverted microscope (Olympus IX-81, UPLFLN 40XO 40 x/1.3 oil-immersion objective), different tomatine concentrations (1-30 µM) were added with a continuous gravity-fed perfusion system. Fluorescence was collected using an sCMO-based imaging system (pco.edge 4.2) running µManager software (Edelstein et al., 2010). Cells were incubated with Fluo-4 (Molecular Probes, 1 μM) as described in (Moreno et al., 2015), and thoroughly washed with an external solution (mM): 100 NaCl, 5 KCl, 2 CaCl_2_, 1 MgCl_2_, 90 sorbitol, 5 glucose, and 10 HEPES (pH 7.4 adjusted with Tris-Base). Fluo-4 loaded cells were excited at 480 nm, and the fluorescence emission at 510 nm was collected and recorded at 2 Hz. At the end of each experiment, ionomycin (1 µM) was added to determine the cell viability (not shown). Signals were recorded, and the background intensity was subtracted using equivalent regions of interest (ROI) outside the cell. Fluo-4 results are expressed as normalized fluorescence (F/F_0_).

### Animals

Immunodeficient NSG (The Jackson Laboratory, Bar Harbor, ME, United States) mice received autoclaved chow and acidified water and ad libitum were bred and housed in individually ventilated cages (NexGenTM, Allentown) under specific pathogen-free conditions and 12:12 photoperiod (less than 325 lux conditions) in The *In vivo* Tumor Biology Research Facility of the Centro Oncológico at the Universidad Católica del Maule (UCM). Animal health was monitored at least three times per week until the experiment ended, following a UCM-approved specific tumor burden scoring system. All procedures were performed in Class II A2 Biological Safety Cabinet. All animal experiments were approved by the Institutional Animal Care and Use Committee of the UCM folio: 2018-03-C, date: 28 June 2018.

### Tumor xenograft assays

5 × 10^6^ HepG2 cells in 100 µL in PBS were injected subcutaneously into the dorsal flank of 5 weeks old NOD scid gamma (NSG) mice under isoflurane anesthesia. All mice were weighed weekly, and tumor growth was monitored by a digital caliper to determine the tumor volume using the formula: (length x width^2^)/2 (Carlsson et al., 1983). Once tumors reached 80-100 mm^3^, mice were randomly assigned to different experimental groups (*n* = 6) and treated by intraperitoneal injection (100 µL) as follows: 5 and 20 mg/kg tomatine, according to previous reports (Lee et al., 2013b; Fan et al., 2014; Ma et al., 2015), and vehicle solution (PBS) twice a week. Mice were monitored daily during the treatment, and weight and tumor volume were determined twice a week.

### Statistical analysis

Statistical values were evaluated by one-way analysis of variance (ANOVA), followed by Dunnett’s multiple comparisons tests and were expressed as mean ± standard error of the mean (SEM). Statistical analysis was performed using GraphPad Prism 8 (GraphPad Software, San Diego, California, United States). *p* < 0.05 was considered statistically significant.

## Results

### Tomatine induced hepatocarcinoma cells cytotoxicity and apoptosis

**FIGURE 1:**
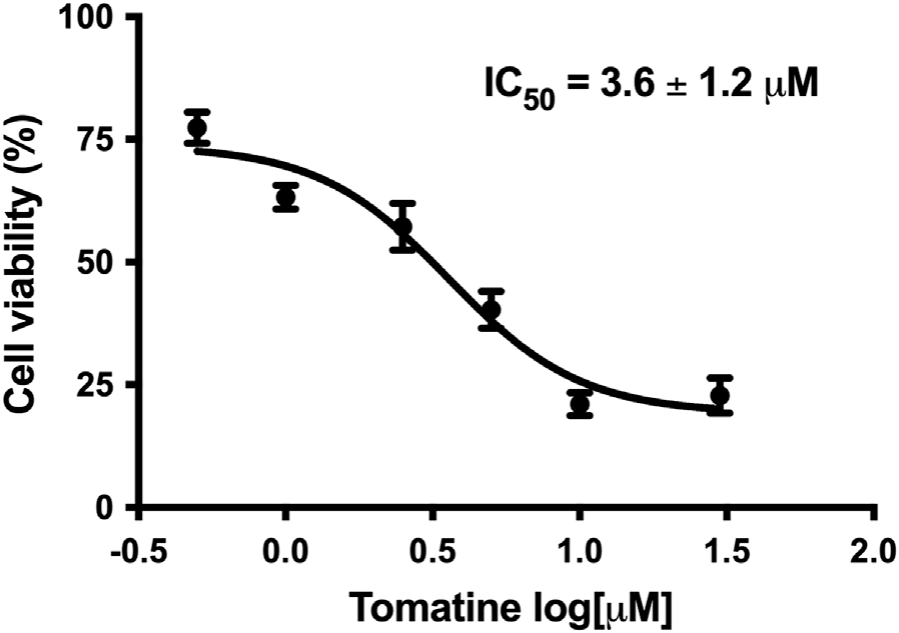
Concentration-response curve of tomatine in HepG2 cells after 24 h incubation as determined with resazurin. Data shown are mean ± SEM of 3 independent experiments.

The treatment of HepG2 cells with increased tomatine concentrations provoked a dose-dependent inhibition of cell proliferation, as determined by the resazurin assay. Significant inhibition of cell viability was noticed at all tested concentrations after 24 h of tomatine treatment. An IC_50_ of around 3.6 ± 1.2 µM for tomatine treatment was obtained (Figure 1) compared to the IC_50_ value of 1.7 ± 1.2 µM of positive control Doxorubicin (DOX) (Supplementary Figure S1).

**FIGURE 2:**
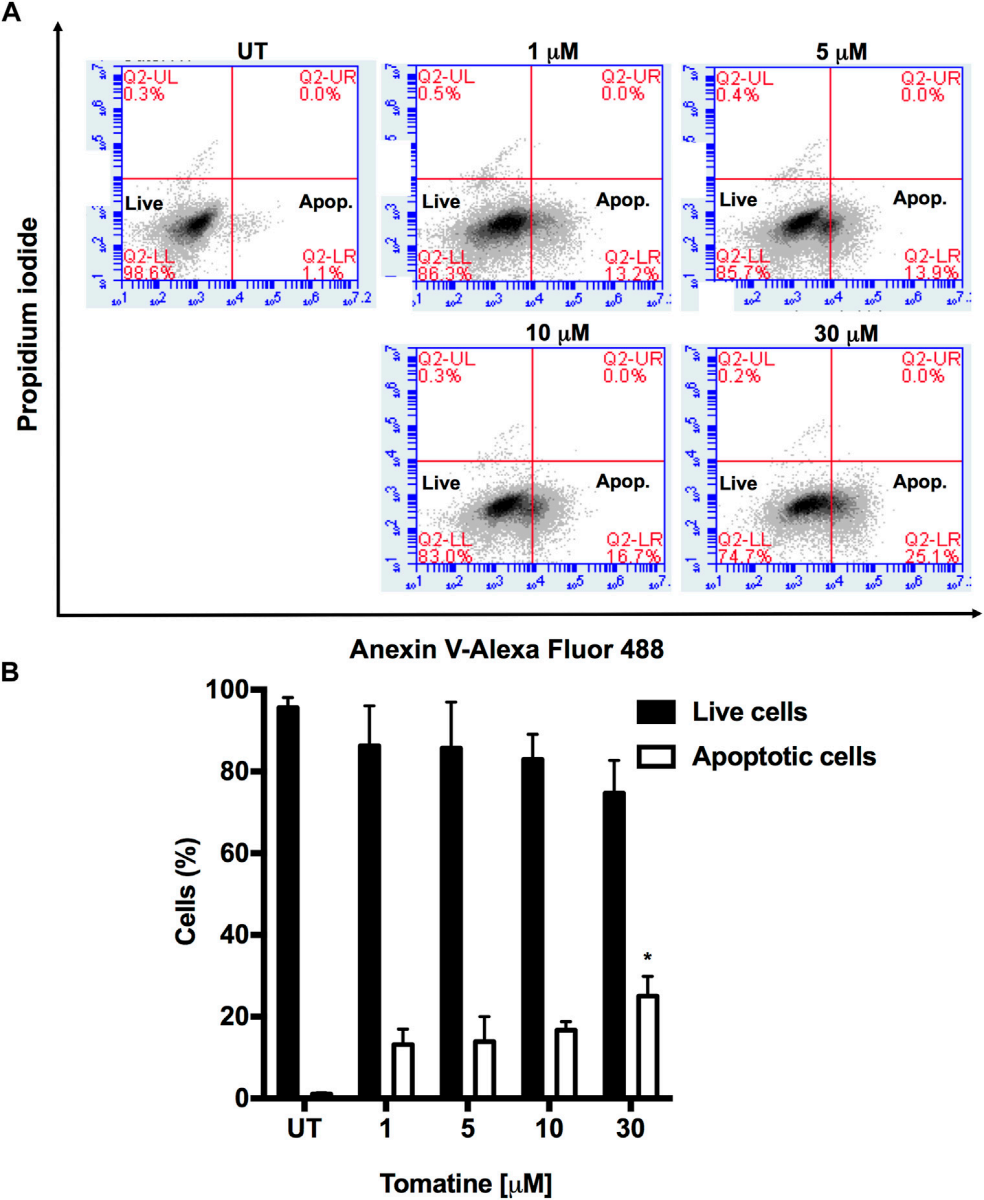
**(A)** Tomatine-induced apoptosis in HepG2 cells as assayed by flow cytometry. HepG2 cells were treated with tomatine (1–30 μM) for 24 h. The cells were then harvested and stained with Annexin V and PI, and flow cytometric analysis was performed to analyze the apoptosis. **(B)** Summary of the apoptosis data in histogram form. *p < 0.05 vs. the untreated (UT) group.

Resistance to apoptosis is one of the hallmarks exhibited by cancer cells, and one of the crucial objectives in cancer treatment is to overcome this resistance via the generation of new drugs to activate the cell death program and involved signaling (Kuno et al., 2012; Ramirez-Tagle et al., 2016). The treatment of HepG2 cells with different tomatine concentrations for 24 h significantly triggered apoptosis, reaching 25.1% at 30 µM of the compound, as shown in Figure 2.

**FIGURE 3:**
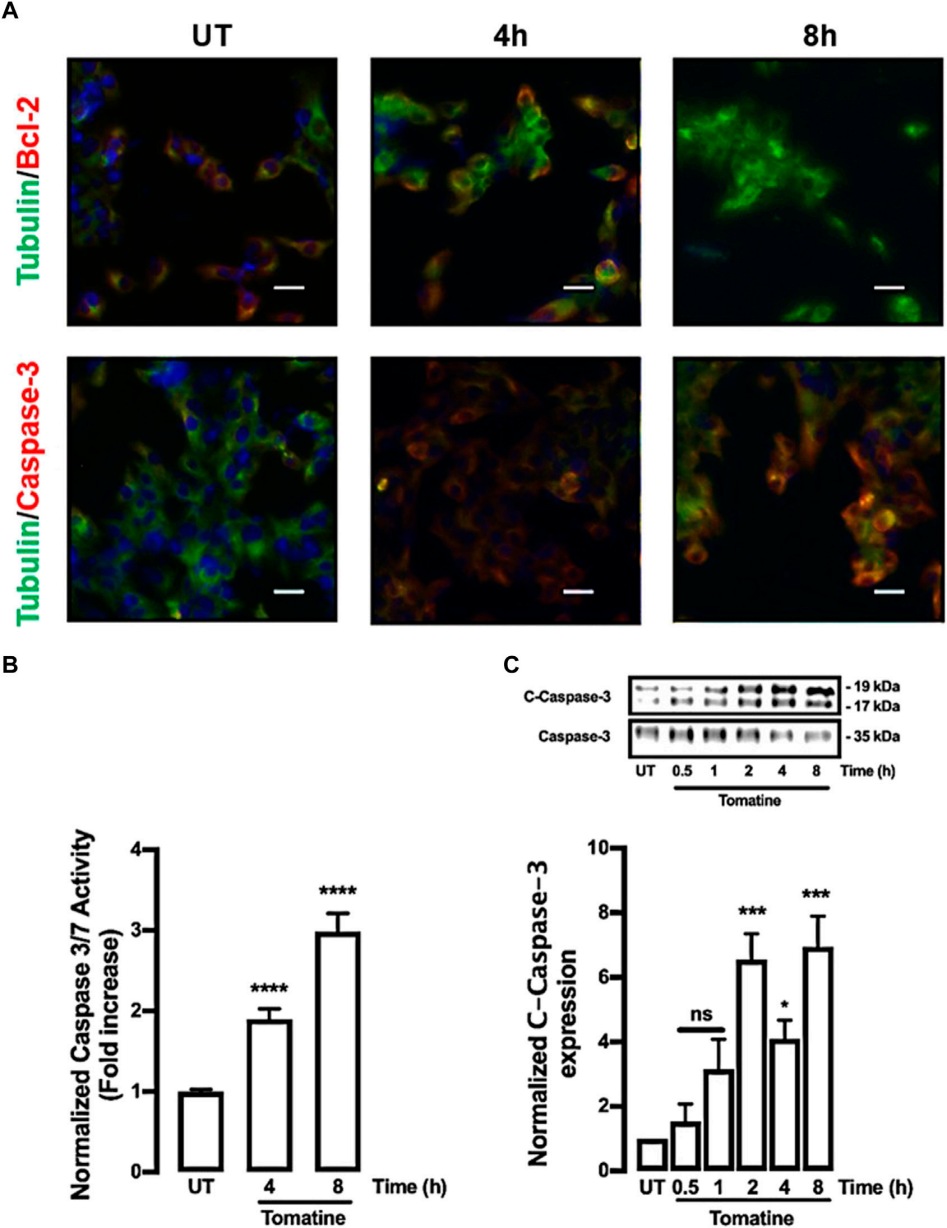
Protein expression of caspase-3, Bcl-2, and activity of caspase 3/7 in HepG2 cells. Cells were treated with 5 μM of tomatine for 4 and 8 h, then immunofluorescence analysis, luminescence, and western blot were performed as described in materials and methods. **(A)** Cells were fixed and immunostained with anti-tubulin antibody (green), anti-caspase-3, or anti-Bcl-2 (red), and cell nuclei were counterstained with DAPI reagent (blue). Tubulin was used to control expression. The bar scale represents 20 μm. **(B)** Caspase Glo Assay results are expressed as a normalized activity from untreated (UT) control cells. **(C)** Western blot analysis of caspase 3. Data shown are mean ± SEM of 3 independent experiments.

Later, it was determined whether tomatine can activate caspases, key cysteine proteases family that regulates apoptosis (Green and Llambi, 2015; Nirmala and Lopus, 2019). Immunofluorescence analysis of cells treated with 5 µM of tomatine for 4 and 8 h increased caspase-3 along with inhibition of the antiapoptotic Bcl-2 protein expression (Figure 3A). Similarly, tomatine strongly induced caspases 3/7 activation (Figure 3B). The increase of caspase activation was also confirmed by western blot analysis revealing the induction, by 5 µM of tomatine treatment, of the procaspase-3 (35 kDa) proteolytic-dependent cleavage to the active caspase-3 (17 kDa) form (Figure 3C) and noticeable between 2 and 8 h of treatment.

### Tomatine induced protein expression alterations of anti-apoptotic and pro-apoptotic pathways

**FIGURE 4:**
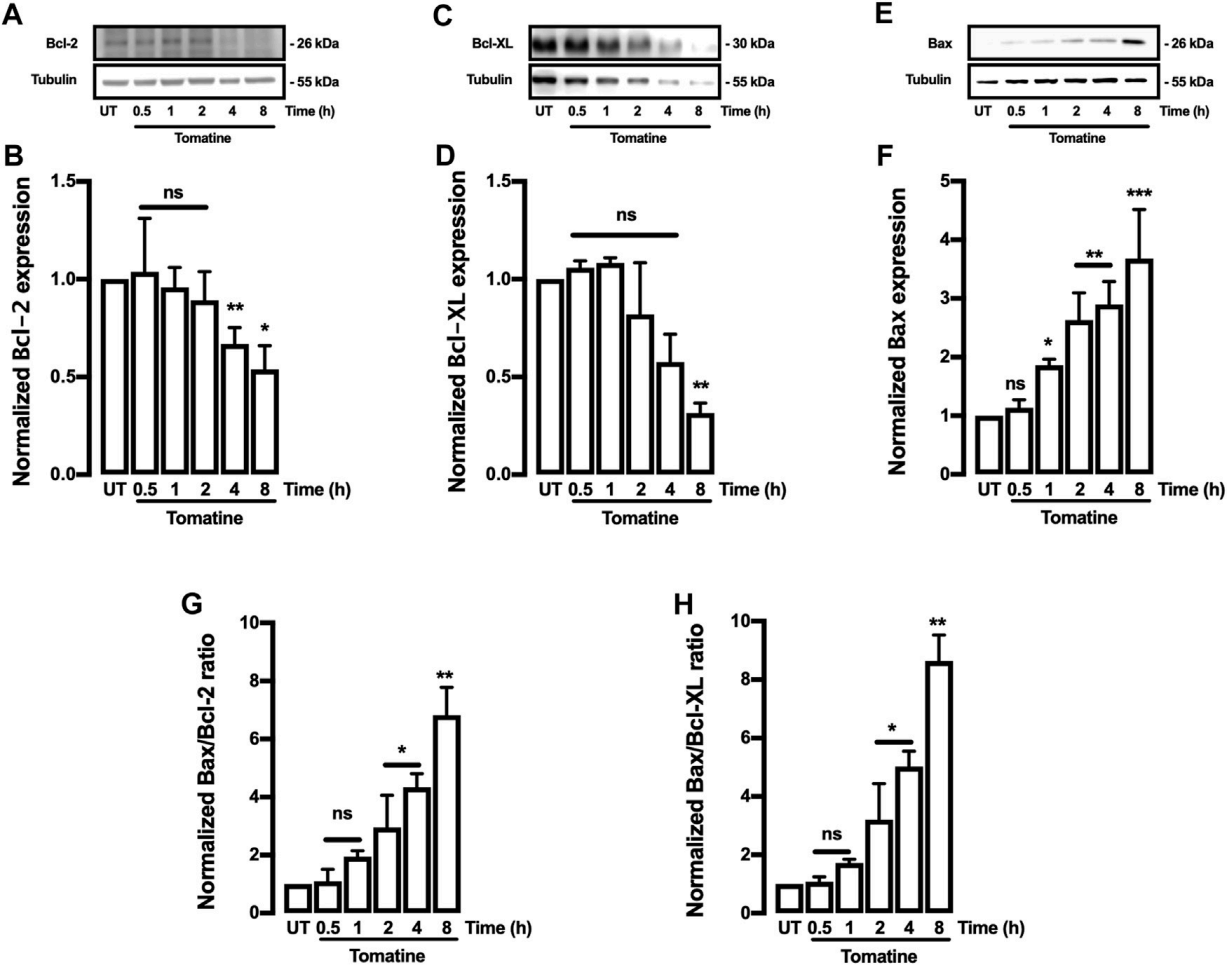
Western blot analysis for Bcl-2, Bcl-XL, and Bax proteins in HepG2 cells. **(A–F)** Cells were treated with tomatine (5 μM), and protein expression was analyzed. **(A,C,and E)** representative images from western blot experiments performed for the detection of proteins. **(B,D,and F)** Densitometric analyses of the experiments are shown in **(A,C,and E)** respectively. **(G,H)** the data were presented in the bar graphs as Bax/Bcl-2 and Bcl-XL ratio. Protein levels were normalized against tubulin and caspase-3. Data are expressed relative to the UT (untreated) condition. Statistical differences were assessed by a one-way ANOVA (Kruskal-Wallis) followed by Dunn’s post hoc test. *p < 0.05; **p < 0.01; ***p < 0.001 vs. untreated (UT) group.

**FIGURE 5:**
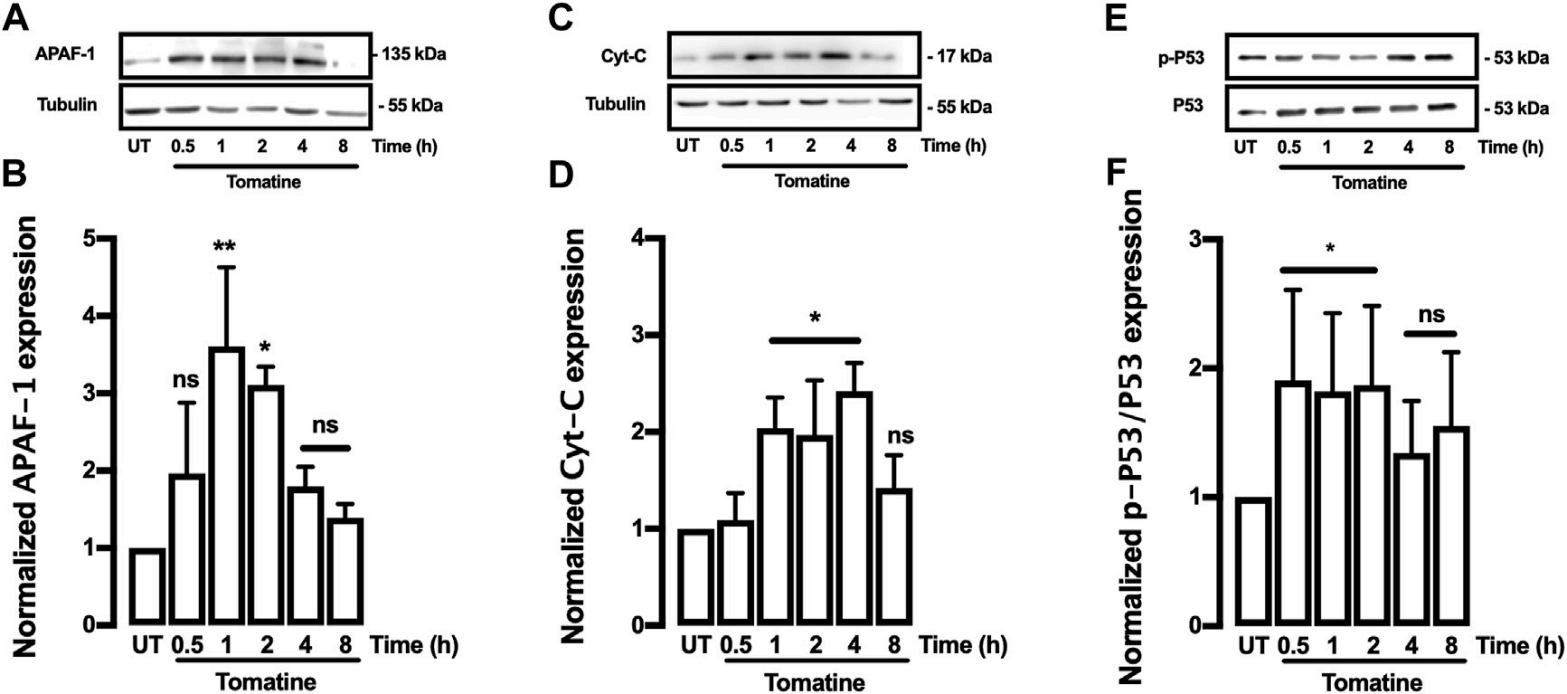
Western blot analysis for APAF-1, Cyt-C, and P53 proteins in HepG2 cells. **(A–F)** Cells were treated with Tomatine (5 μM), and protein expression was analyzed. **(A,C, and E)** representative images from western blot experiments performed for the detection of proteins. **(B,D, and F)** Densitometric analyses of the experiments are shown in **(A,C, and E)** respectively. Protein levels were normalized against tubulin, and caspase-3 and data are expressed relative to the untreated (UT) condition. Statistical differences were assessed by a one-way ANOVA (Kruskal-Wallis) followed by Dunn’s post hoc test. *p < 0.05; **p < 0.01; ***p < 0.001 vs. UT group.

The effect of tomatine on the expression of apoptosis-related proteins such as family-Bcl-2 proteins like Bcl-2 and Bax was evaluated. The Bcl-2, Bcl-XL, Cytochrome-c (Cyt-c), and Bax proteins are associated with antiapoptotic and pro-apoptotic functions. As shown in Figures 4A,B, tomatine treatment at 5 μM significantly decreased Bcl-2 expression in contrast to the levels of the control cells at 4 and 8 h; similarly, treatment with tomatine significantly decreased Bcl-XL expression after 8 h (Figures 4C,D). Conversely, Bax protein expression increased significantly at 2, 4, and 8 h after treatment with tomatine (Figures 4E,F). Densitometric analysis of tomatine revealed a significant increase of Bax/Bcl-2 and Bax/Bcl-xl ratio at 2-8 h (Figures 4G,H). Next, it was determined the role of apoptotic protease-activating factor 1 (Apaf-1), a key molecule for the activation of caspase-9 in the apoptosome. Apaf-1 protein expression, increased significantly at 1 and 2 h after treatment with tomatine (Figures 5A,B).

Subsequently, its was investigated whether tomatine promotes leakage of the mitochondrial intermembrane content such as Cyt-c. Western blot analysis showed that Cyt-c release significantly in HepG2 cells treated with 5 μM tomatine after increase 1 until 4 h (Figures 5C,D). Finally, to determine whether Bcl-2 and Bcl-XL downregulation depend on the p53 pathway, tomatine’s effect on the p53 activation was examined. Tomatine treatment led to a time-dependent induction of phosphorylated P53 (p-P53) (Figures 5E,F), with p-P53 protein being upregulated in tomatine-treated cells within 30 min until 2 h of treatment, while after 4 and 8 h of treatment no significant increase was observed. These results indicate that tomatine-induced apoptosis was mediated *via* a p53-dependent pathway, consistent with studies in other glycoalkaloids (Ding et al., 2013).

### Tomatine treatment-induced intracellular ROS generation in HepG2 cells

**FIGURE 6:**
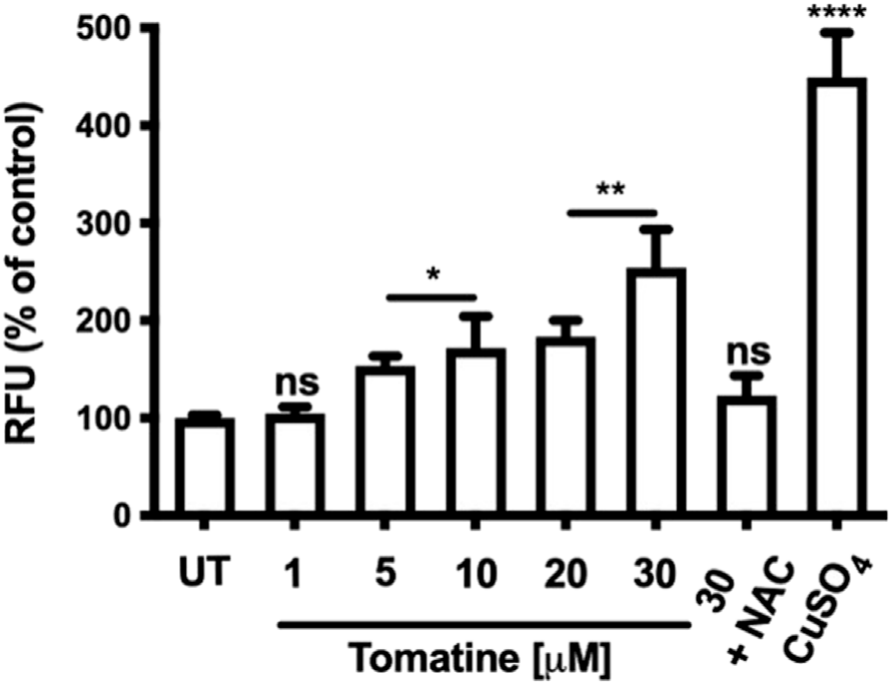
Effects of tomatine on HepG2 cells intracellular ROS generation. Cells were exposed to tomatine at 1-30 μM for 4 h and co-treated with NAC. CuSo_4_ is used as a positive control. Stained cells with DCFDA and analyzed by a fluorescence plate reader. Data are expressed as the mean ± SEM from three independent experiments, each performed in triplicate. A one-way ANOVA (Kruskal–Wallis) assessed statistical differences, followed by Dunn’s post hoc test. *p < 0.05; **p < 0.01; ****p < 0.001 vs. Untreated (UT) group. The bar scale represents 20 μm.

ROS generation has been implicated as an early event in apoptosis. Then, it was determined whether tomatine induced ROS in HepG2 cells. In cells incubated with 1-30 μM of tomatine 4 h before fluorescence analysis (Figure 6), a rapid and significant generation of ROS was detected at 5 μM concentration of tomatine, reaching a maximum of 30 μM with a value 250% higher than that in control cells. Pre-incubation with NAC 5 mM prevented the increase of ROS in HepG2 cells. CuSO_4_ used as a positive control.

### Tomatine-evoked intracellular Ca^2+^ rises in HepG2 cells

**FIGURE 7:**
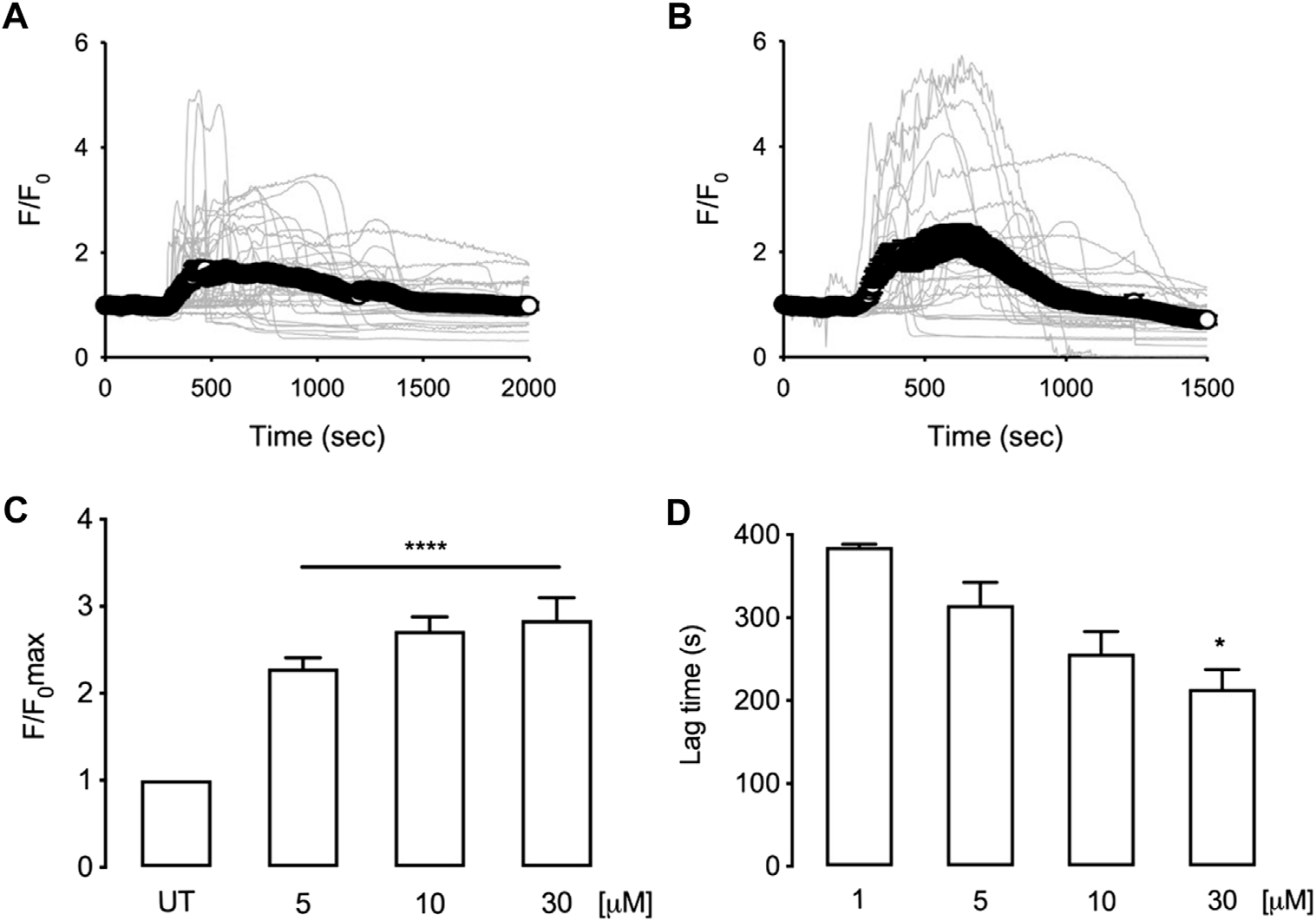
Time courses for normalized fluorescence in HepG2 cells loaded with Fluo4 and treated with 5 µM **(A)** or 30 µM **(B)** tomatine. Grey lines correspond to [Ca^2+^]_i_ changes in individual cells (40 cells, 6 independent experiments for 5 μM; 27 cells, 5 independent experiments for 30 µM). White circles correspond to the mean trace of [Ca^2+^] signals. Bar graphs show the summary of maximal normalized fluorescence **(C)** or response lag time **(D)** obtained from experiments as shown in **(A)** and **(B)** data are shown as mean ± sem. A one-way ANOVA (Kruskal–Wallis) assessed statistical differences, followed by Dunn’s post hoc test. *p < 0.05; **p < 0.01; ****p < 0.001 vs. Untreated (UT) group.

Figures 7A,B illustrate the intracellular calcium recorded in HepG2 cells treated with 5 or 30 µM of tomatine. As shown, even 5 µM of tomatine induces a transient intracellular calcium increase within the first 15 min; moreover, the maximal recorded fluorescent signal does not change much with increasing doses (Figure 7C). However, cells exposed to increasing doses of tomatine (Figure 7D) show a faster response lag time (defined as the time where the temporal derivative of the fluorescence exceeds 10% of its maximum value). These results suggest that in HepG2 cells, intracellular stores are the origin of the observed intracellular calcium increase as previously shown in neuroblastomas (da Silva et al., 2017).

### Tomatine attenuated HepG2 growth of cell xenograft tumors in mice

**FIGURE 8:**
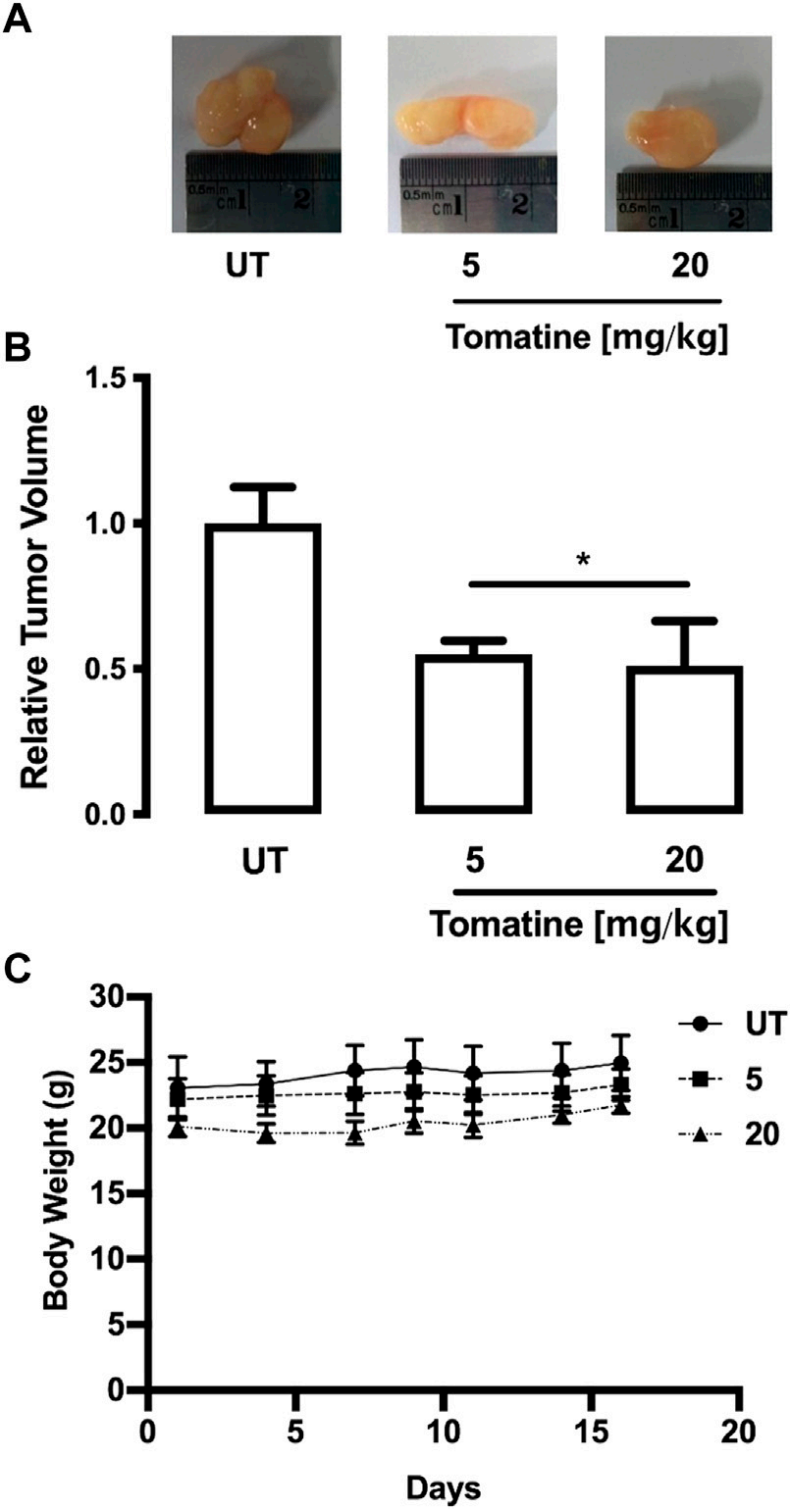
HepG2 tumor growth was inhibited by tomatine treatment in a xenograft mouse model. **(A)** Representative images of tumors from each group treatment in hepatoma xenograft tumor growth. **(B)** Tumor volumes of HepG2 xenografts during the 3-weeks treatment period for tomatine and saline control. *p < 0.05 for tomatine vs. saline control group (*n* = 6 each). **(C)** Representative body weight curve of mice bearing HepG2 xenografts during the 3-weeks treatment period with tomatine. Statistical differences were assessed by a one-way ANOVA (Kruskal–Wallis) followed by Dunn’s post hoc test. *p < 0.05 vs. untreated (UT) group.

To examine the effect of tomatine on tumor growth *in vivo*, HepG2 cell subcutaneous tumors grown in mice were challenged. In these experiments, tumors were allowed to establish for 1 week before challenge 3 times per week for 3 weeks with tomatine (5 or 20 mg/kg/mouse) and vehicle solution. Figure 8A shows that 5 and 20 mg/kg tomatine were equally efficient at retarding the growth of HepG2 cell subcutaneous mouse tumors. Specifically, 3 weeks after the commencement of the drug challenge, the relative tumor volume in-vehicle control compared to mice treated with 5 and 20 mg/kg tomatine was about half compared to untreated animals (Figure 8B). Besides, tomatine treatment did not provoke changes in the body weight of mice (Figure 8C).

## Discussion

Tomatine has demonstrated promising anticancer function on solid tumors (Lee et al., 2004; Ramirez-Tagle et al., 2016). Still, the underlying biological and molecular mechanisms have not been well elucidated yet. The results of this investigation indicated that it inhibited the viability of the HCC cancer cell line HepG2 *in vitro* in a dose-dependent fashion (Figure 1). In this sense, the results agree with had been reported in several other cancer cell lines (Friedman et al., 2009; Huang et al., 2015). The inhibition of cell proliferation was accompanied by apoptotic induction, partly due to caspase-3 activation, Bcl2 downregulation, and increased activation of the tumor suppressor protein p53. Specifically, P53 downregulates Bcl2 expression at the transcriptional level by interaction with its promoter in the regulatory binding sequence (Wu et al., 2001).

Besides the induction of cell cycle arrest, tomatine can also inhibit tumor cell proliferation via induction of apoptosis (Chao et al., 2012; Kim et al., 2015). ROS have a crucial role in induced apoptosis (Simon et al., 2000; Redza-Dutordoir and Averill-Bates, 2016). Increases in intracellular ROS promote the mitochondrial permeability transition pore (mPTP) opening, thus promoting transitory permeabilization of the mitochondrial inner membrane, mainly contributing to apoptosis activation. Indeed, the extrinsic and intrinsic apoptosis pathways load the mPTP (Kinnally et al., 2011). The treatment with tomatine for 4 h significantly increased intracellular ROS levels (Figure 6) consistently with the notion of ROS as an early response mechanism during apoptosis induction (Merad-Boudia et al., 1998; Ni et al., 2008).

Furthermore, the integrity of the outer mitochondrial membrane depends on the balance of the anti- and pro-apoptotic Bcl-2 family proteins expression (Raisova et al., 2001; Breckenridge and Xue, 2004). Tomatine downregulated the antiapoptotic Bcl-2 and Bcl-xl protein expression while upregulating Bax and Bad expression, both playing a role as pro-apoptotic factors. Therefore, tomatine disrupted the antiapoptotic-proapoptotic balance in favor of cell death. Indeed, Bax/Bcl-2 and Bax/Bcl-xl ratios were significantly increased after 4-8 h of tomatine treatment, which may enhance cells’ susceptibility to death signals and mitochondrial function in response to the treatment (Raisova et al., 2001). In addition, the activation of Bax due to tomatine treatment may indicate that apoptosis induction can be mediated by the endoplasmic reticulum (ER) stress pathway in HepG2 cells. Besides, one of the characteristics of many antitumoral agents is their capacity to increase ROS levels causing an oxidative stress activation of apoptosis (Lau et al., 2008). Drugs that induce oxidative stress result in lipid peroxidation and antioxidant defenses reduction, such as reduced glutathione levels, superoxide dismutase, catalase, and thioredoxin expressions, which highly contribute to the apoptosis induction program (Yang et al., 2008). Moreover, for the expression of cell death programs in mammalian cells, the mitochondrial-dependent apoptotic pathway is critical in chemotherapy to kill cancer cells (Polčic and Mentel, 2020). In this sense, a Bax/Bcl-2 ratio increase is critical in the apoptosis process because it stimulates the release of cytochrome c (Cyt-c) from mitochondria into the cytosol (Kirkin et al., 2004). Cyt-c interacts with Apaf-1 to form the apoptosome–deoxyadenosine triphosphate-dependent complex, and the apoptosis program is initiated with the activation of caspase-3 and caspase-9 (Zhang, 2019). Consistently, on HepG2 cells, tomatine provoked a dysregulation of Bax/Bcl-2 ratio, increased Cyt-c cytoplasmic release and apaf-1 expression alongside caspase-3 and caspase-7 activation.

P53 plays a key role in ROS-promoted DNA damage response and apoptosis induction (Maillet and Pervaiz, 2012). Hence, in HepG2 cells, tomatine promoted p53 activation and Bcl-2 reduction with subsequent activation of mitochondrial downstream molecular pathways such as caspase-7 and caspase-3 activation. These results agree with previous investigations demonstrating that ROS may induce DNA damage along with p53 activation in tumoral development (Maillet and Pervaiz, 2012; Budanov, 2014; Li et al., 2020).

Calcium signaling is involved in various cellular functions, including cell proliferation and apoptotic cell death (Berridge et al., 2003). Furthermore, the disruption of intracellular calcium homeostasis can induce ER stress and, consequently, cellular apoptosis (Wang et al., 2013; Monteith et al., 2017). Our results show that tomatine induces a transient increase of intracellular calcium within 15 min. Increasing doses of tomatine reduce the response time instead of augmenting the amount of calcium change, suggesting that internal calcium stores are involved in the mechanism of action tomatine. This observation agrees with previous reports that show, in SH-SY5Y cells, that this alkaloid induces an increase in the basal level of calcium after 1 h of incubation, probably because of ER stress (da Silva et al., 2017), the kinetics of calcium increase also suggests that the increase of calcium could be one of the first steps in the observed tomatine-induced apoptosis insert paragraph. Other glycoalkaloids have been studied for their ability to induce apoptosis; a group of them are present in *Solanum melongena* L. fruit peels have promising anticancer activity against HCC (Maha M Salama, 2013), but there is no evidence on the mechanism by which tomatine induces apoptosis in HCC. ROS and calcium together can initiate apoptosis signals in various cell types and are crucial candidates to initiate apoptosis in HCC by tomatine treatment (Wang et al., 2003; Maha M Salama, 2013).

Besides, tomatine also demonstrated *in vivo* inhibitory capacity of tumor HepG2 cell growth. The IP injection of 5 and 20 mg/kg body weight of tomatine significantly reduced tumor sizes. The concentrations of the antitumor compound seem not toxic as no major-organs abnormalities and non-loss of the animals’ body weight were produced (Figure 8). Thus, the results suggest that tomatine can be safely used in tumor animal models at the experimental conditions used in this study and consistently with the agent’s concentrations used in previous investigations (Chao et al., 2012; Lee et al., 2013b; Kim et al., 2015). Therefore, tomatine demonstrated its potential use as a chemopreventive and chemotherapeutic agent for future clinical strategies in cancer patients.

Interestingly, the tomatine’s antiproliferative and promoting cell death activities can be explained by its capacity to downregulate several intracellular signals associated with these events. Tomatine inhibits ERK, PI3K/AKT, and NF-κB pathways that can be implicated in inhibiting HepG2 cell proliferation, apoptotic induction, and *in vivo* tumor growth (Shih et al., 2009; Lee et al., 2013b; Bailly, 2021). Moreover, recently the epidermal growth factor receptor (EGFR) has been indicated as a direct tomatine molecular target acting as receptor tyrosine kinase inhibitor by a molecular docking into ATP binding sites of EGFR. In this sense, HepG2 cells express EGFR and depend on the EGF/EGFR signal transduction for proliferation and metastatic capabilities (Huang et al., 2014; Chen et al., 2021). Nevertheless, further experimental analyses are necessary to determine the potential contribution of EGFR inhibition in the capacity of tomatine to inhibit *in vitro* and *in vivo* tumoral functions of HepG2 cells.

Tomatine inhibits cancer cell proliferation and *in vivo* tumor growth of cancer cells, such as prostate (Lee et al., 2013a), solid Ehrlich tumor (Tomsik et al., 2013), and HepG2 (this study), and in several cancer cell lines *in vitro* as well (Xiuwei et al., 2007; Choi et al., 2012; Shi et al., 2013; Del Giudice et al., 2015; Huang et al., 2015; Kim et al., 2015; Rudolf and Rudolf, 2016). This study may have limitations because it was conducted with only one cell line. Nevertheless, we expect to confirm our results with other HCC and non-tumorigenic hepatic cell lines in further experiments, which may increase the impact of our findings.

**FIGURE 9:**
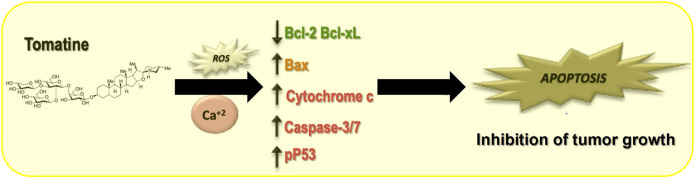
Proposed model for tomatine-mediated apoptosis in human hepatoma cells.

Despite the mentioned limitation, this study contributes to a better understanding of the molecular and biological mechanisms of tomatine implicated in its antitumoral functions. Next, we propose a simplified model indicating the involved mechanisms in tomatine inhibition of HCC (Figure 9).

On the safe use in humans, several studies have shown that tomatine is not toxic when consumed orally in moderate amounts (Friedman et al., 2007). For example, Peruvians consume a tomato variant very rich in tomatine (0.5–5 mg/g dry weight) without presenting visible signs of toxicity (Rick et al., 1994). In mice, LD_50_ values of 1000, 500, 25–33.5 and 18 mg/kg body weight have been reported for α-tomatine by subcutaneous, oral, intraperitoneal and intravenous routes, respectively (Wilson et al., 1961; Friedman, 2002). All these data contribute to the evidence for the safe therapeutic use of tomatine in humans.

## Conclusion

In conclusion, tomatine potently inhibited the HCC HepG2 cell line viability by inducing cellular apoptosis, which involved the increased intracellular ROS and calcium, p53 activation along with Bcl-2 downregulation, and mitochondrial-dependent caspase cascade activation. Also, this glycoalkaloid agent inhibited *in vivo* HepG2 tumor growth in a SCID mouse xenograft model. Together, the current investigation results indicate that tomatine is an excellent therapeutic candidate for treating human HCC.

## Data Availability

The original contributions presented in the study are included in the article/Supplementary Material, further inquiries can be directed to the corresponding authors.

## Abbreviations

Apaf-1, apoptotic protease-activating factor 1; Bax, BCL2 Associated X; Bcl-2, B-cell CLL/lymphoma-2; Cyt-c, Cytochrome c; DBP, dibenzo[a,l]pyrene; DCFH-DA, 2,7-Dichlorodihydrofluorescein diacetate; DMEM, Dulbecco’s modified Eagle Medium; ER, endoplasmic-reticulum; FBS, fetal bovine serum; HCC, hepatocellular carcinoma; IVC, individually ventilated cages; NSG, NOD scid gamma; PBS, Phosphate-buffered saline; ROS, reactive oxygen species; RFU, relative fluorescence unity’s; sCMO, stationary camera with moving objects.

## References

[B1] Bailly C. (2021). The steroidal alkaloids α-tomatine and tomatidine: Panorama of their mode of action and pharmacological properties. Steroids 176, 108933. 10.1016/j.steroids.2021.108933 34695457

[B2] Berridge M. J. Bootman M. D. Roderick H. L. (2003). Calcium signalling: Dynamics, homeostasis and remodelling. Nat. Rev. Mol. Cell Biol. 4, 517–529. 10.1038/nrm1155 12838335

[B3] Breckenridge D. G. Xue D. (2004). Regulation of mitochondrial membrane permeabilization by BCL-2 family proteins and caspases. Curr. Opin. Cell Biol. 16, 647–652. 10.1016/j.ceb.2004.09.009 15530776

[B4] Budanov A. V. (2014). The role of tumor suppressor p53 in the antioxidant defense and metabolism. Subcell. Biochem. 85, 337–358. 10.1007/978-94-017-9211-0_18 25201203 PMC4206257

[B5] Carlsson G. Gullberg B. Hafström L. (1983). Estimation of liver tumor volume using different formulas---An experimental study in rats. J. Cancer Res. Clin. Oncol. 105, 20–23. 10.1007/BF00391826 6833336 PMC12252809

[B6] Chao M.-W. Chen C.-H. Chang Y.-L. Teng C.-M. Pan S.-L. (2012). α-Tomatine-mediated anti-cancer activity *in vitro* and *in vivo* through cell cycle-and caspase-independent pathways. PLoS One 7, e44093. 10.1371/journal.pone.0044093 22970166 PMC3435411

[B7] Chen H. Wu X. Zhou H. He Z. Li H. Wang Q. (2021). Epidermal growth factor upregulates the expression of A20 in hepatic cells via the MEK1/MSK1/p-p65 (Ser276) signaling pathway. Am. J. Transl. Res. 13, 708–718. 33594320 PMC7868826

[B8] Choi S. H. Ahn J.-B. Kozukue N. Kim H.-J. Nishitani Y. Zhang L. (2012). Structure–activity relationships of α-β1-γ-and δ-tomatine and tomatidine against human breast (MDA-MB-231), gastric (KATO-III), and prostate (PC3) cancer cells. J. Agric. Food Chem. 60, 3891–3899. 10.1021/jf3003027 22482398

[B9] da Silva D. C. Andrade P. B. Valentao P. Pereira D. M. (2017). Neurotoxicity of the steroidal alkaloids tomatine and tomatidine is RIP1 kinase-and caspase-independent and involves the eIF2α branch of the endoplasmic reticulum. J. Steroid Biochem. Mol. Biol. 171, 178–186. 10.1016/j.jsbmb.2017.03.009 28300624

[B10] Del Giudice R. Raiola A. Tenore G. C. Frusciante L. Barone A. Monti D. M. (2015). Antioxidant bioactive compounds in tomato fruits at different ripening stages and their effects on normal and cancer cells. J. Funct. Foods 18, 83–94. 10.1016/j.jff.2015.06.060

[B11] Ding X. Zhu F. Yang Y. Li M. (2013). Purification, antitumor activity *in vitro* of steroidal glycoalkaloids from black nightshade (Solanum nigrum L). Food Chem. 141, 1181–1186. 10.1016/j.foodchem.2013.03.062 23790901

[B12] Distl M. Wink M. (2009). Identification and quantification of steroidal alkaloids from wild tuber-bearing Solanum species by HPLC and LC-ESI-MS. Potato Res. 52, 79–104. 10.1007/s11540-008-9123-0

[B13] Echeverría C. Montorfano I. Cabello-Verrugio C. Armisén R. Varela D. Simon F. (2015). Suppression of transient receptor potential melastatin 4 expression promotes conversion of endothelial cells into fibroblasts via transforming growth factor/activin receptor-like kinase 5 pathway. J. Hypertens. 33, 981–992. 10.1097/HJH.0000000000000496 25909699

[B14] Edelstein A. Amodaj N. Hoover K. Vale R. Stuurman N. (2010). Computer control of microscopes using µManager. Curr. Protoc. Mol. Biol. 92, Unit14.20. 10.1002/0471142727.mb1420s92 PMC3065365 20890901

[B15] Fan C. Zheng W. Fu X. Li X. Wong Y.-S. Chen T. (2014). Strategy to enhance the therapeutic effect of doxorubicin in human hepatocellular carcinoma by selenocystine, a synergistic agent that regulates the ROS-mediated signaling. Oncotarget 5, 2853–2863. 10.18632/oncotarget.1854 24797310 PMC4058050

[B16] Faria-Silva C. de Sousa M. Carvalheiro M. C. Simões P. Simões S. (2022). Alpha-tomatine and the two sides of the same coin: An anti-nutritional glycoalkaloid with potential in human health. Food Chem. 391, 133261. 10.1016/j.foodchem.2022.133261 35640336

[B18] Friedman M. (2013). Anticarcinogenic, cardioprotective, and other health benefits of tomato compounds lycopene, α-tomatine, and tomatidine in pure form and in fresh and processed tomatoes. J. Agric. Food Chem. 61, 9534–9550. 10.1021/jf402654e 24079774

[B19] Friedman M. Levin C. E. Lee S.-U. Kim H.-J. Lee I.-S. Byun J.-O. (2009). Tomatine-containing green tomato extracts inhibit growth of human breast, colon, liver, and stomach cancer cells. J. Agric. Food Chem. 57, 5727–5733. 10.1021/jf900364j 19514731

[B20] Friedman M. McQuistan T. Hendricks J. D. Pereira C. Bailey G. S. (2007). Protective effect of dietary tomatine against dibenzo [a, l] pyrene (DBP)‐induced liver and stomach tumors in rainbow trout. Mol. Nutr. Food Res. 51, 1485–1491. 10.1002/mnfr.200700176 17979099

[B21] Friedman M. (2002). Tomato glycoalkaloids: Role in the plant and in the diet. J. Agric. Food Chem. 50, 5751–5780. 10.1021/jf020560c 12358437

[B22] Green D. R. Llambi F. (2015). Cell death signaling. Cold Spring Harb. Perspect. Biol. 7, a006080. 10.1101/cshperspect.a006080 26626938 PMC4665079

[B23] Gregory P. Sinden S. L. Osman S. F. Tingey W. M. Chessin D. A. (1981). Glycoalkaloids of wild, tuber-bearing Solanum species. J. Agric. Food Chem. 29, 1212–1215. 10.1021/jf00108a028

[B24] Huang H. Chen S. Van Doren J. Li D. Farichon C. He Y. (2015). α-Tomatine inhibits growth and induces apoptosis in HL-60 human myeloid leukemia cells. Mol. Med. Rep. 11, 4573–4578. 10.3892/mmr.2015.3238 25625536 PMC4735690

[B25] Huang P. Xu X. Wang L. Zhu B. Wang X. Xia J. (2014). The role of EGF ‐ EGFR signalling pathway in hepatocellular carcinoma inflammatory microenvironment. J. Cell. Mol. Med. 18, 218–230. 10.1111/jcmm.12153 24268047 PMC3930409

[B26] Kaunda J. S. Zhang Y.-J. (2019). The genus Solanum: An ethnopharmacological, phytochemical and biological properties review. Nat. Prod. Bioprospect. 9, 77–137. 10.1007/s13659-019-0201-6 30868423 PMC6426945

[B27] Kim S. P. Nam S. H. Friedman M. (2015). The tomato glycoalkaloid α-tomatine induces caspase-independent cell death in mouse colon cancer CT-26 cells and transplanted tumors in mice. J. Agric. Food Chem. 63, 1142–1150. 10.1021/jf5040288 25614934

[B28] Kinnally K. W. Peixoto P. M. Ryu S.-Y. Dejean L. M. (2011). Is mPTP the gatekeeper for necrosis, apoptosis, or both? Biochim. Biophys. Acta 1813, 616–622. 10.1016/j.bbamcr.2010.09.013 20888866 PMC3050112

[B29] Kirkin V. Joos S. Zörnig M. (2004). The role of Bcl-2 family members in tumorigenesis. Biochim. Biophys. Acta 1644, 229–249. 10.1016/j.bbamcr.2003.08.009 14996506

[B30] Kulik L. El-Serag H. B. (2019). Epidemiology and management of hepatocellular carcinoma. Gastroenterology 156, 477–491. 10.1053/j.gastro.2018.08.065 30367835 PMC6340716

[B31] Kuno T. Tsukamoto T. Hara A. Tanaka T. (2012). Cancer chemoprevention through the induction of apoptosis by natural compounds. J. Biophys. Chem. 3, 156–173. 10.4236/jbpc.2012.32018

[B32] Lau A. T. Y. Wang Y. Chiu J. (2008). Reactive oxygen species: Current knowledge and applications in cancer research and therapeutic. J. Cell. Biochem. 104, 657–667. 10.1002/jcb.21655 18172854

[B33] Lee K. R. Kozukue N. Han J. S. Park J. H. Chang E. Y. Baek E. J. (2004). Glycoalkaloids and metabolites inhibit the growth of human colon (HT29) and liver (HepG2) cancer cells. J. Agric. Food Chem. 52, 2832–2839. 10.1021/jf030526d 15137822

[B34] Lee S.-T. Wong P.-F. He H. Hooper J. D. Mustafa M. R. (2022). Correction: Alpha-Tomatine attenuation of *in vivo* growth of subcutaneous and orthotopic xenograft tumors of human prostate carcinoma PC-3 cells is accompanied by inactivation of nuclear factor-kappa B signaling. PLoS One 17, e0268234. 10.1371/journal.pone.0268234 35503799 PMC9064074

[B35] Lee S.-T. Wong P.-F. Hooper J. D. Mustafa M. R. (2013a). Alpha-tomatine synergises with paclitaxel to enhance apoptosis of androgen-independent human prostate cancer PC-3 cells *in vitro* and *in vivo* . Phytomedicine 20, 1297–1305. 10.1016/j.phymed.2013.07.002 23920276

[B36] Lee S. T. Wong P. F. He H. Da Hooper J. Mustafa M. R. (2013b). Alpha-tomatine attenuation of *in vivo* growth of subcutaneous and orthotopic xenograft tumors of human prostate carcinoma PC-3 cells is accompanied by inactivation of nuclear factor-kappa B signaling. PLoS One 8, e57708. 10.1371/journal.pone.0057708 23437404 PMC3578807

[B37] Li Y. Jiang B. Wang R. Wang J. Li Y. Bao Y. (2020). Synergistic effects of tanshinone IIA and andrographolide on the apoptosis of cancer cells via crosstalk between p53 and reactive oxygen species pathways. Pharmacol. Rep. 72, 400–417. 10.1007/s43440-019-00006-z 32048269

[B38] Ma L. Wang X. Jia T. Wei W. Chua M.-S. So S. (2015). Tankyrase inhibitors attenuate WNT/β-catenin signaling and inhibit growth of hepatocellular carcinoma cells. Oncotarget 6, 25390–25401. 10.18632/oncotarget.4455 26246473 PMC4694839

[B39] Maha M Salama M. M. S. (2013). *In vitro* and *in vivo* anticancer activity of the fruit peels of Solanum melongena L. Against hepatocellular carcinoma. J. Carcinog. Mutagen. 4, 149–154. 10.4172/2157-2518.1000149

[B40] Maillet A. Pervaiz S. (2012). Redox regulation of p53, redox effectors regulated by p53: A subtle balance. Antioxid. Redox Signal. 16, 1285–1294. 10.1089/ars.2011.4434 22117613

[B41] Merad-Boudia M. Nicole A. Santiard-Baron D. Saillé C. Ceballos-Picot I. (1998). Mitochondrial impairment as an early event in the process of apoptosis induced by glutathione depletion in neuronal cells: Relevance to Parkinson’s disease. Biochem. Pharmacol. 56, 645–655. 10.1016/S0006-2952(97)00647-3 9783733

[B42] Monteith G. R. Prevarskaya N. Roberts-Thomson S. J. (2017). The calcium-cancer signalling nexus. Nat. Rev. Cancer 17, 367–380. 10.1038/nrc.2017.18 28386091

[B43] Moreno C. Hermosilla T. Morales D. Encina M. Torres-Díaz L. Díaz P. (2015). Cavβ2 transcription start site variants modulate calcium handling in newborn rat cardiomyocytes. Pflugers Arch. 467, 2473–2484. 10.1007/s00424-015-1723-3 26265381

[B44] Ni Y. Gong X. Lu M. Chen H. Wang Y. (2008). Mitochondrial ROS burst as an early sign in sarsasapogenin-induced apoptosis in HepG2 cells. Cell Biol. Int. 32, 337–343. 10.1016/j.cellbi.2007.12.004 18262806

[B45] Nirmala J. G. Lopus M. (2019). Cell death mechanisms in eukaryotes. Cell Biol. Toxicol. 36, 145–164. 10.1007/s10565-019-09496-2 31820165

[B46] Osman S. F. Herb S. F. Fitzpatrick T. J. Schmiediche P. (1978). Glycoalkaloid composition of wild and cultivated tuber-bearing Solanum species of potential value in potato breeding programs. J. Agric. Food Chem. 26, 1246–1248. 10.1021/jf60219a024

[B47] Polčic P. Mentel M. (2020). Reconstituting the mammalian apoptotic switch in yeast. Genes 11, 145. 10.3390/genes11020145 PMC7073680 32013249

[B48] Raisova M. Hossini A. M. Eberle J. Riebeling C. Orfanos C. E. Geilen C. C. (2001). The Bax/Bcl-2 ratio determines the susceptibility of human melanoma cells to CD95/Fas-mediated apoptosis. J. Invest. Dermatol. 117, 333–340. 10.1046/j.0022-202x.2001.01409.x 11511312

[B49] Ramirez-Tagle R. Escobar C. Romero V. Montorfano I. Armisén R. Borgna V. (2016). Chalcone-induced apoptosis through caspase-dependent intrinsic pathways in human hepatocellular carcinoma cells. Int. J. Mol. Sci. 17, 260. 10.3390/ijms17020260 26907262 PMC4783989

[B50] Redza-Dutordoir M. Averill-Bates D. A. (2016). Activation of apoptosis signalling pathways by reactive oxygen species. Biochim. Biophys. Acta 1863, 2977–2992. 10.1016/j.bbamcr.2016.09.012 27646922

[B51] Rick C. M. Uhlig J. W. Jones A. D. (1994). High alpha-tomatine content in ripe fruit of andean Lycopersicon esculentum var. cerasiforme: Developmental and genetic aspects. Proc. Natl. Acad. Sci. U. S. A. 91, 12877–12881. 10.1073/pnas.91.26.12877 7809139 PMC45543

[B52] Riss T. L. Moravec R. A. Niles A. L. Minor L. (2017). in Cell viability assays. Editors Gilbert D. F. Friedrich O. (New York, NY: Springer New York). 10.1007/978-1-4939-6960-9

[B53] Rudolf K. Rudolf E. (2016). Antiproliferative effects of α-tomatine are associated with different cell death modalities in human colon cancer cells. J. Funct. Foods 27, 491–502. 10.1016/j.jff.2016.10.005

[B54] Shi M.-D. Shih Y.-W. Lee Y.-S. Cheng Y.-F. Tsai L.-Y. (2013). Suppression of 12-O-Tetradecanoylphorbol-13-Acetate-Induced MCF-7 breast adenocarcinoma cells invasion/migration by α-tomatine through activating PKCα/ERK/NF-κB-Dependent MMP-2/MMP-9 expressions. Cell biochem. Biophys. 66, 161–174. 10.1007/s12013-012-9465-8 23114726

[B55] Shih Y.-W. Shieh J.-M. Wu P.-F. Lee Y.-C. Chen Y.-Z. Chiang T.-A. (2009). Alpha-tomatine inactivates PI3K/akt and ERK signaling pathways in human lung adenocarcinoma A549 cells: Effect on metastasis. Food Chem. Toxicol. 47, 1985–1995. 10.1016/j.fct.2009.05.011 19457446

[B56] Simon H. U. Haj-Yehia A. Levi-Schaffer F. (2000). Role of reactive oxygen species (ROS) in apoptosis induction. Apoptosis 5, 415–418. 10.1023/A:1009616228304 11256882

[B57] Tomsik P. Micuda S. Sucha L. Cermakova E. Suba P. Zivny P. (2013). The anticancer activity of alpha-tomatine against mammary adenocarcinoma in mice. Biomed. Pap. Med. Fac. Univ. Palacky. Olomouc Czech. Repub. 157, 153–161. 10.5507/bp.2013.031 23681309

[B58] Van Gelder W. M. J. (1985). Determination of the total C27-steroidal alkaloid composition of Solanum species by high-resolution gas chromatography. J. Chromatogr. A 331, 285–293. 10.1016/0021-9673(85)80034-0

[B59] Wang C. Liu C. Niu L. Wang L. Hou L. Cao X. (2013). Surfactin-induced apoptosis through ROS-ERS-Ca2+-ERK pathways in HepG2 cells. Cell biochem. Biophys. 67, 1433–1439. 10.1007/s12013-013-9676-7 23733672 PMC3838591

[B60] Wang H. Yang X. Zhang Z. Xu H. (2003). Both calcium and ROS as common signals mediate Na2SeO 3-induced apoptosis in SW480 human colonic carcinoma cells. J. Inorg. Biochem. 97, 221–230. 10.1016/S0162-0134(03)00284-8 14512201

[B61] Wilson R. H. Poley G. W. DeEds F. (1961). Some pharmacologic and toxicologic properties of tomatine and its derivatives. Toxicol. Appl. Pharmacol. 3, 39–48. 10.1016/0041-008x(61)90006-0 13785704

[B62] Wu H. Li W. Wang T. Rong Y. He Z. Huang S. (2021). α-Tomatine, a novel early-stage autophagy inhibitor, inhibits autophagy to enhance apoptosis via Beclin-1 in Skov3 cells. Fitoterapia 152, 104911. 10.1016/j.fitote.2021.104911 33901572

[B63] Wu Y. Mehew J. W. Heckman C. A. Arcinas M. Boxer L. M. (2001). Negative regulation of bcl-2 expression by p53 in hematopoietic cells. Oncogene 20, 240–251. 10.1038/sj.onc.1204067 11313951

[B64] Xiuwei Y. Fuxiang R. Ruiqing W. Jun W. Jun P. Huiping P. (2007). Inhibitory effects of 44 alkaloids compounds against growth of human gastric carcinoma cell line BGC and human hepatic carcinoma cell line BEL-7402 *in vitro* [J]. Mod. Chin. Med. 2.

[B65] Yang H.-L. Chen S.-C. Chen C.-S. Wang S.-Y. Hseu Y.-C. (2008). Alpinia pricei rhizome extracts induce apoptosis of human carcinoma KB cells via a mitochondria-dependent apoptotic pathway. Food Chem. Toxicol. 46, 3318–3324. 10.1016/j.fct.2008.08.003 18768154

[B66] Zhang T. M. (2019). TRIAP1 inhibition activates the cytochrome c/Apaf-1/Caspase-9 signaling pathway to enhance human ovarian cancer sensitivity to cisplatin. Chemotherapy 64, 119–128. 10.1159/000501633 31661694

