# Supplementary material for: In Vivo and in vitro antitumor activity of tomatine in hepatocellular carcinoma
Source: Front Pharmacol. 2022 Sep 9;13:1003264. doi: 10.3389/fphar.2022.1003264 (PMC9501894; doi:10.3389/fphar.2022.1003264)
Supplement: Supplementary file 1 [file Table1.DOCX]

# **Table S1.** Primary and secondary antibodies used in western blot experiments

| Primary Ab | *Dilution* | *Source* | *Incubation*  *time* | *Incubation*  *temperature* | *Brand* |
| --- | --- | --- | --- | --- | --- |
| Bax | 1:500 | Rabbit | 2 h | RT | Santa Cruz, CA, USA |
| Bcl-2 | 1:500 | Rabbit | 2 h | RT | Santa Cruz, CA, USA |
| Caspase-3 | 1:1000 | Rabbit | 2 h | RT | Cell Signaling, MA, USA |
| Bcl-XL | 1:1000 | Rabbit | 2 h | RT | Abcam, Cambridge, UK |
| Caspase-9 | 1:1000 | Mouse | 2 h | RT | Cell Signaling, MA, USA |
| HSP70 | 1:5000 | Mouse | 2 h | RT | Origene, MD, USA |
| α-Tubulin | 1:5000 | Mouse | 2 h | RT | Sigma-Aldrich, MO, USA |
| APAF-1 | 1:1000 | Rabbit | 2 h | RT | Abcam, Cambridge, UK |
| Cyt-C | 1:1000 | Rabbit | 2 h | RT | Abcam, Cambridge, UK |
| P53 | 1:1000 | Mouse | 2 h | RT | Cell Signaling, MA, USA |
| p-P53 | 1:1000 | Mouse | 2 h | RT | Cell Signaling, MA, USA |

| Secondary Ab | *Dilution* | *Source* | *Incubation*  *time* | *Incubation*  *temperature* | *Brand* |
| --- | --- | --- | --- | --- | --- |
| Anti- Mouse HRP | 1:5000 | Goat | 2 h | RT | Millipore, MA, USA |
| Anti- Rabbit HRP | 1:5000 | Goat | 2 h | RT | Millipore, MA, USA |

Ab: antibody, RT: room temperature, ON: overnight
