# Supplementary material for: In Vivo and in vitro antitumor activity of tomatine in hepatocellular carcinoma
Source: Front Pharmacol. 2022 Sep 9;13:1003264. doi: 10.3389/fphar.2022.1003264 (PMC9501894; doi:10.3389/fphar.2022.1003264)
Supplement: Supplementary file 2 [file Table2.DOCX]

# **Table S2.** Primary and secondary antibodies used in immunocytochemistry.

## Immunocytochemistry

| Primary Ab | *Dilution* | *Source* | *Incubation*  *time* | *Incubation*  *temperature* | *Brand* |
| --- | --- | --- | --- | --- | --- |
| Bcl-2 | 1:100 | Rabbit | ON | 4ºC | Santa Cruz, CA, USA Santa Cruz, CA, USA |
| Caspase-3 | 1:100 | Rabbit | ON | 4ºC | Cell Signaling, MA, USA |
| α-Tubulin | 1:500 | Mouse | ON | 4ºC | Sigma-Aldrich, MO, USA |

| Secondary Ab | *Dilution* | *Source* | *Incubation*  *time* | *Incubation*  *temperature* | *Brand* |
| --- | --- | --- | --- | --- | --- |
| Anti- Rabbit Alexa-594 | 1:250 | Goat | ON | 4ºC | Invitrogen, CA, USA  USA |
| Anti- Mouse Alexa-488 | 1:250 | Goat | ON | 4ºC | Invitrogen, CA, USA |

Ab: antibody, RT: room temperature, ON: over night
