# Supplementary material for: In Vivo and in vitro antitumor activity of tomatine in hepatocellular carcinoma
Source: Front Pharmacol. 2022 Sep 9;13:1003264. doi: 10.3389/fphar.2022.1003264 (PMC9501894; doi:10.3389/fphar.2022.1003264)
Supplement: Supplementary file 3 [file Image1.pdf]

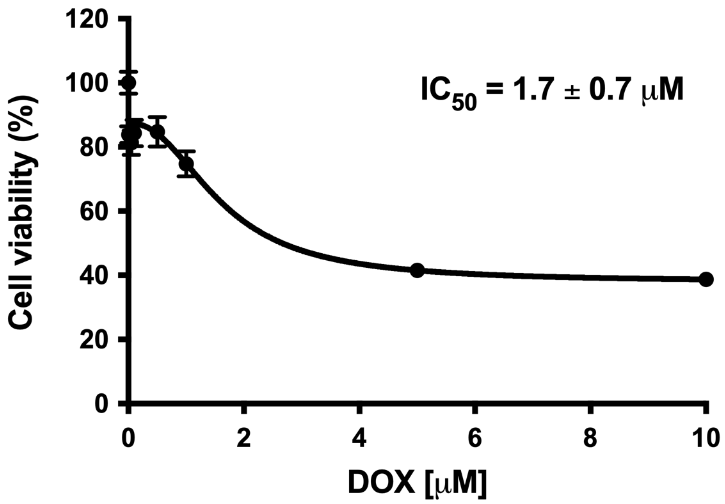

Figure S1. Concentration-response curve of positive control Doxorubicin (DOX) in HepG2 cells after 24 h incubation as determined with resazurin. Data shown are mean ± SEM of 3 independent experiments.
